# The Role of Intracellular Organisms in the Pathogenesis of Inflammatory Arthritis

**DOI:** 10.1155/2014/158793

**Published:** 2014-06-05

**Authors:** Animesh Singh, Sarah Karrar

**Affiliations:** Department of Rheumatology, Charing Cross Hospital, Imperial College NHS Trust, London W6 8RF, UK

## Abstract

Inflammatory arthritis is a condition which is characterised by recurrent episodes of joint pain and swelling. It encompasses a spectrum of disorders ranging from rheumatoid arthritis to ankylosing spondylitis. In these conditions, for reasons that are poorly understood, the immune system raises an inflammatory response within the joint space. In some cases, autoantigens have been identified (e.g., anticitrullinated peptides in rheumatoid arthritis), but the absence of these, in the seronegative arthritides, for example, raises question as to the underlying pathogenesis. Interest has, therefore, turned to host-pathogen interactions and whether aberrant immune responses to these could explain the development of arthritis. This has been most widely studied in reactive arthritis (ReA), where an infectious episode precedes the development of the joint symptoms. In this review, we present the evidence for the role of host-bacterial interactions in the pathogenesis of joint inflammation with particular emphasis on ReA. We discuss a range of possible mechanisms including molecular mimicry, persistent low grade infections, and abnormal host responses to common bacterial causes of reactive arthritis as well as discussing some of the clinical challenges that we face in making the diagnosis and in treatment of persistent symptoms.

## 1. Introduction

Infectious organisms are increasingly being recognised as an important contributor to inflammatory arthritides. This has encompassed diseases ranging from chronic lower back pain to rheumatoid arthritis [1, 2]. In this review we will concentrate on the role of intracellular pathogens in reactive arthritis (ReA), which has been most widely studied.

ReA belongs to the family of spondyloarthritides that share features of inflammatory lower back pain, oligoarthritis, and extra-articular symptoms. Despite the widespread use of the term, there remains no universal classification or diagnostic criteria for ReA. The original definition involved the development of a sterile and inflammatory arthritis during or following an infection elsewhere in the body. Subsequently microbial antigenic material and, in some cases, replicating microbes were found in joints although cultures were negative. In 1999 the 4th International Workshop on Reactive Arthritis discussed the use of the term ReA and proposed its use if the clinical picture and microbes involved are associated with HLA-B27 and spondyloarthritis while “infection-related arthritis” should be used with other arthritides related to infections [3]. Classification criteria proposed by the workshop are shown below (Box 1) although clinical use is limited by availability of synovial biopsies for polymerase-chain reaction (PCR) and immunohistology.

The classic bacteria associated with ReA are Gram-negative obligate or facultative intracellular aerobic bacteria with a lipopolysaccharide-containing outer membrane. The primary focus of infection is thought to be through the mucus membrane, either in the gut or in the urogenital tract.

Gastrointestinal microbes commonly include* Salmonella*,* Shigella*, and* Campylobacter jejuni*. Less frequently diagnosed infections include* Campylobacter lari*,* Chlamydia psittaci*, and* Clostridium difficile*.* Chlamydia trachomatis* is the most common cause of genital infection and the most common triggering infection for ReA.

## 2. Molecular Mimicry and Persistent Infection

Molecular mimicry refers to sequence similarities between pathogen derived antigens and human or host native peptides such that a cross-reaction occurs. This results in autoantibodies being formed against self-antigens and an inappropriate inflammatory response with subsequent tissue damage.

In ReA, molecular mimicry provides a “neat” explanation of how the immune system can lose tolerance to host tissue following an episode of infection. It is feasible that infection with specific pathogens primes the immune system to recognise and therefore attack certain host antigens. Studies looking at sequence homology between pathogens associated with reactive arthritis and human peptides have identified several possible candidate genes.

Infection with* Chlamydia trachomatis *is the most common worldwide cause of reactive arthritis. Between 4 and 8% of patients with sexually transmitted chlamydial infections develop reactive arthritis. Persistence of* Chlamydia* in host tissue results in chronic infection [4, 5]. Infection in the genitourinal tract has been associated with increased risk of ReA. More recent data has also shown that the bacteria persist in the joint itself in patients with ReA [4–6]. Several studies have demonstrated the presence of* Chlamydia* species via microscopy and PCR [6–8]. It is thought that once infected via the respiratory or genital tract,* Chlamydia* species can invade not only local epithelial cells but also local monocytes and macrophages [4]. These then carry the bacteria via the blood stream to the synovial membrane where they become embedded. Here, they exist in a unique state, inducing an ongoing inflammatory response without causing an outright suppurative infection. During its normal infection cycle,* Chlamydia* (in its elementary body form) is taken up by the host cell (in the case of monocytes by phagocytosis) [4, 8]. Between 12 and 24 hours later, the pathogen morphs into its active form known as the reticulate body and starts to multiply within the host cell, eventually being released back to disseminate the infection. In the persistent state associated with ReA, the* Chlamydia* displays features at variance with its normal cycle. In particular, there is downregulation of genes associated with replication and host cell MHC expression, allowing the organism to avoid immune destruction [8, 9]. A persistent inflammatory response is maintained by infected monocytes that secrete proinflammatory cytokines such as interferon-*γ* (IFN-*γ*), interleukin 1 (IL-1), and tumour necrosis factor (TNF) [8]. This leads to a local reaction involving the joint causing pain and swelling.

Persistent forms of infection expose the host continuously to bacterial antigens and it has been postulated that some of these antigens cross-react with host cells leading to inflammation and damage. In one study, homology between* Chlamydia* DNA primase and several sequences in the human genome has been found and interestingly all are human leukocyte antigen (HLA) B27 restricted [9]. The corresponding proteins and the exact role of these DNA sequences in the pathogenesis of the disease have yet to be identified.

The incidence of ReA following* Salmonella* gastroenteritis has been estimated as 12 per 1,000 cases of infection [4]. ReA patients have stronger and more prolonged antibody response than those who do not develop joint symptoms [10]. In addition, bacterial components seem to persist in both the peripheral blood cells and the joint itself [11]. Both DNA and* Salmonella* lipopolysaccharide have been identified in synovial fluid and the synovial membrane of patients [11, 12]. It is thought that these persistent components provide antigenic material driving an ongoing immune response. One suggested mechanism is through molecular mimicry and several studies have identified candidate antigens. One such protein is the OmpH outer membrane protein which has homology with the alpha helix of the HLA-B27 molecule, which forms part of the important antigen binding site [13]. In an early study, sera from patients with ReA reacted to synthetic versions of OmpH [13]. Interestingly, further data obtained from studying patients with enthesitis related juvenile idiopathic arthritis (JIA) found that these patients' peripheral blood mononuclear cells had an antigen specific proliferative response to OmpH [14].

ReA following* Shigella* infection is rare with an estimated incidence rate of 1 per million in the developed world [15]. This may in part be due to the scarcity of this infection in these areas as once the infection is acquired the incidence seems to be 12 per 1,000 cases of* Shigella* gastroenteritis [16]. As with* Salmonella*,* Shigella* DNA has been found in synovial fluid of patients with undifferentiated spondyloarthropathy although the clinical significance of this is not clear [17]. Studies between arthrogenic and nonarthrogenic strains of* Shigella* have illuminated several potential pathogenic mechanisms. Supportive evidence for molecular mimicry is tenuous. Comparisons between* S. flexneri* and* S. Sonnei* (the former of which is arthrogenic while the latter is not) have identified several molecular differences including 2 proteins which share homology with HLA-B27. However, this remains inconclusive and reports of ReA following infection with classically nonarthrogenic strains suggest that this may not be a significant pathogenic pathway [15].

*Yersinia* associated ReA was first reported in the late 1960s. The exact incidence is difficult to ascertain due to difficulties in making the diagnosis. Several studies have demonstrated the presence of* Yersinia* DNA in the synovial fluid of patients with ReA and undifferentiated spondyloarthropathy. The presence of bacteria or bacterial components has thus been theorised as propagating the inflammatory response.

There is tenuous evidence for the role of molecular mimicry in the pathogenesis of* Yersinia* associated arthritis. Homology between several* Yersinia* proteins and HLA-B27 has been identified [13]. One such bacterial protein candidate is the Yad A protein which is an adhesion molecule. This shares homology with the antigen binding site of HLA-B27 [13, 18]. However, although studies have demonstrated that patients with ReA and spondyloarthropathy can recognise these proteins, they have failed to show that patient sera bind to HLA-B27 and, in fact, patients' antibodies tend to recognise the nonhomologous portions of the bacterial substrates [13, 18].

## 3. Mouse Models: What Do They Tell Us about Reactive Arthritis?

The established animal model for reactive arthritis is the* Chlamydia trachomatis* induced arthritis model (CtIA), in which intra-articular* Chlamydia* species are used to provoke inflammatory arthropathy [19]. These animals developed an acute monoarthritis in the first 48 hours after injection which is severe and destructive, likely representing an acute septic arthritis. By day 21 the animals have a persistent arthritis which is aseptic and is considered to be a model of reactive arthritis [19]. Interestingly, although whole bacteria were not found, persistence of certain antigens has been demonstrated in joint tissue including outer membrane protein (MOMP) and* Chlamydia* lipopolysaccharide which are thought to perpetuate the inflammatory response [19]. Comparison between different rat strains to CtIA showed that susceptible strains had altered clearance of bacteria [20] from the joint with organisms persisting longer in synovial samples. Interestingly, susceptible Lewis strain of rat had reduced local production of IFN-*γ* and TNF*α* which mirrors findings found in patients with reactive arthritis where an imbalance of Th1/Th2 cytokines is thought to contribute to poor organism clearance (see above). Interestingly, other experimental studies have found that deficiency of TNF*α* was associated with more severe infection and more severe arthritis [21].

Existing models for other inflammatory arthritides tend to fall into 2 categories—spontaneous and induced. In the former, murine strains develop arthritis spontaneously and are used generally to identify genetic components to disease susceptibility and underlying pathogenic mechanisms. In spontaneous models, mice or rats are selectively bred if they develop an inflammatory arthropathy and genetic association studied, for example, in the MRL/lpr mouse [22, 23]. Most of these mouse models develop an autoimmune arthritis that clinically and histologically resembles rheumatoid arthritis. In alternative spontaneous models, transgenic mice are created to look at the role of specific molecules in the development of inflammatory arthropathy, for example, mice overexpressing TNF-alpha [23] who spontaneously developed arthritis which clinically and histopathologically corresponded with rheumatoid arthritis in humans.

In the induced models of inflammatory arthritis, mouse strains known to be susceptible are injected with an immunostimulant to induce arthritis. The first such model was the injection of Freund's adjuvant into susceptible rats which then developed an acute arthritis [24]. Similar disease phenotype could also be induced using adjuvants which lack immunogenic material such as incomplete Freund's adjuvant, avridine, and pristine [25]. The typical course in these animal models is an acute onset severe arthritis which is frequently self-limiting unlike the spontaneous models which often have a chronic and remitting course. Similarity with seronegative arthropathy and reactive arthritis lies in the abrupt onset and frequent large joint oligoarthritis. Unlike seronegative arthropathy, however, these animals have not been shown to develop axial disease nor enthesitis. In these models, the disease is thought to be T-cell driven and a delayed hypersensitivity type reaction to the adjuvant and they do not develop a humoral response [26, 27]. In contrast, in human reactive arthritis, as mentioned previously, Th1/Th2 dysregulation is thought to be important in the persistence of bacteria but not directly responsible for joint damage [28–33].

Other induced models of inflammatory arthritis include immunisation of animals with collagen and adjuvant (collagen induced arthritis—CIA) to induce autoantibodies and break self-tolerance. Unlike reactive arthritis, this disease model depends on the development of autoantibodies and histologically resembles rheumatoid arthritis. Interestingly, in some murine CIA models, animals develop an enthesitis which is usually a feature of seronegative spondyloarthropathy. In proteoglycan-induced arthritis, up to 70% of mice developed a spondyloarthropathy [34]. However, unlike human disease, there is autoantibody production as well as rheumatoid factor deposition in joints [34]. Other studies have demonstrated that even transfer of sera can induce disease in previously unimmunised mice, showing an important autoimmune, antibody mediated component to pathogenesis not seen in reactive arthritis.

## 4. Host Susceptibility Factors

A major part in resolving infections, particularly involving intracellular bacteria, is played by cytokines such as IFN-*γ* [35]. In ReA, the antibacterial Th1 cytokine response (IFN-*γ*, IL-2, and IL-12) is impaired in favour of a Th2 response (IL-10 and IL-4) allowing microbes to survive [28–33]. A variety of genetic factors are implied in this Th1/Th2 dysregulation including the polymorphism of host cytokine genes. Data from Finnish patients suggests that the microsatellites IL10.G10 and IL10.G12 from the promoter region of the IL-10 gene seem to be protective against the development of ReA [36]. A German study has demonstrated that the level of TNF*α* secretion by T cells at ReA onset is inversely proportional to the disease duration and severity [37]. It is unlikely that cytokine production and polymorphism can wholly explain the development of ReA and other susceptibility factors certainly play a part. This includes the modulation of HLA-B27 and the lymphocyte response by arthritogenic bacteria such as* Yersinia* and* Salmonella* [38].

Up to 70% of patients with ReA and 50% of those with undifferentiated spondyloarthropathy are HLA-B27 positive. The HLA super-locus is a genomic region in the chromosomal position 6p21 that encodes the six classical transplantation HLA genes and at least 132 protein coding genes that have important roles in the regulation of the immune system. This small segment of the human genome has been associated with more than 100 different diseases, including rheumatoid arthritis, psoriasis, and various other autoimmune disorders [39].

HLA-B27 binds and presents peptides from influenza, HIV, Epstein-Barr virus, and other viruses. This leads to vigorous and specific cytotoxic T lymphocyte responses that play an important role in the body's immune response to these viruses [40]. As it is a MHC class I molecule, it has been theorised that it may have altered antigen presentation properties which renders host cells either more susceptible to infection with arthrogenic pathogens or less effective at clearing infection. Structurally, the molecule differs from other HLA molecules due to slow folding heavy chains, which form aberrant dimers [41, 42]. It is thought that these aberrant dimers accumulate within cells leading to increased unfolded protein response (UPR) that activates the stress response in cells [43, 44]. Markers of the UPR were associated with increased exposure to proinflammatory cytokines such as IFN-*γ* [43]. This suggests that there may be an altered intracellular milieu in HLA-B27 expressing cells that might explain the ability for certain organisms to persist.

Several studies have demonstrated that immune cells obtained from HLA-B27 positive individuals have impaired ability to clear intracellular organisms [45–47]. For example, in one study, HLA-B27 transfected monocytes were more permissive of* Salmonella* replication as compared to those transfected with HLA-A2 controls [47]. One suggested mechanism is reduced nitric oxide production in HLA-B27 expressing cells leading to reduced elimination [45]. Since persistence of organisms has been suggested as an important pathogenic mechanism, these studies may in part explain the increased rate of reactive arthritis in HLA-B27 positive individuals. These findings have not been replicated with* Chlamydia* species so it is likely that this plays a less important role than initially anticipated [48].

As mentioned previously, HLA-B27 has been suggested as a potential target for molecular mimicry. Homology between the HLA-B27 molecule and various bacterial proteins has been found, with conflicting evidence for the significance of this in the pathogenesis.

## 5. Investigation

The investigation of a ReA involves a comprehensive history and examination. There is usually a delay of between 1 and 6 weeks between the start of infection and onset of arthritis. The disease is typically an asymmetric oligoarthritis involving the large joints, dactylitis, and extra-articular features [49].

Besides a typical clinical picture, an appropriate organism needs identification through either isolation or raised antibody levels. It is often possible for enteric microbe isolation during the acute phase of illness from stool samples. However, gastrointestinal symptoms often settle before the development of arthritis and serological diagnosis is required.

Urethritis can be a sign of infection or an extra-articular disease manifestation. Genital infections from* Chlamydia* are often asymptomatic and therefore clinical suspicion is required for appropriate testing. Urine PCR is more convenient than urogenital swabs and offers comparable results. The use of serology for* Chlamydia trachomatis* diagnosis is impaired by high prevalence of positive antibodies amongst the general population together with cross-reactivity with antibodies against* Chlamydia pneumoniae*.

Conventional blood tests including erythrocyte sedimentation rate (ESR) and C-reactive protein (CRP) together with autoantibodies should be performed. The use of HLA-B27 as a diagnostic tool is disputed, as it is often negative in people with mild to moderate disease [50]. Synovial fluid joint aspirates should be performed and typically show leucocytosis. Cultures are usually negative and fluid should be analysed under light microscopy for the presence of crystals.

## 6. Treatment and Outcome

Patients with acute Chlamydia infection should have antibiotic treatment according to local guidelines (e.g., azithromycin 2 gm) and importantly partners also require treatment. There is recent evidence that prolonged courses of antibiotics may be beneficial in the treatment of chronic arthritis associated with persistent Chlamydia infections [51]. Antibiotics are usually not required for uncomplicated enteritis as the infection is often self-limiting and interestingly treatment with antibiotics does not shorten the duration of ReA [52].

Nonsteroidal anti-inflammatories (NSAIDS) are the mainstay of symptomatic control. Intra-articular glucocorticoid injections are also beneficial in patients with mono- or oligoarthritis and systemic steroids are used in refractory cases or in patients with polyarticular disease. Sulfasalazine has been used successfully in chronic cases of reactive arthritis, and early introduction (<3 months) has been shown to be more effective than placebo in inducing remission [53]. There are case reports regarding the successful use of anti-TNF therapy in cases not responding to conventional therapy, particularly in HLA-B27 positive patients [54].

The overall outcome for patients is good with 50% recovering within 6 months and only 15% having a prolonged arthritis beyond 1 year. Long term followup of these patients indicates that 2–18% develop a chronic arthritis, 14–49% sacroiliitis, and 12–26% ankylosing spondylitis [55].

## 7. Conclusion

**Figure 1:**
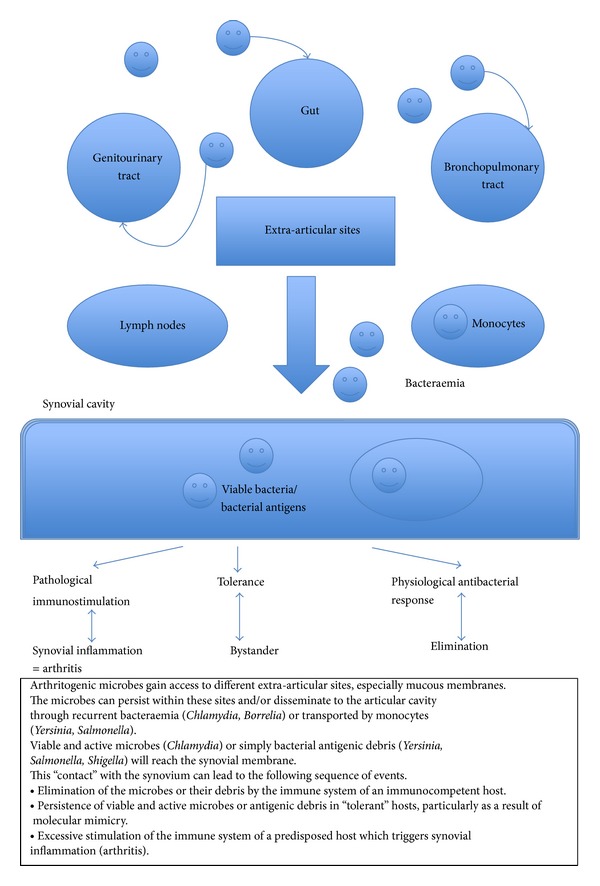
Proposed natural history of reactive arthritis [49].

Host-bacterial interactions play an essential role in the pathogenesis of ReA. A combination of bacterial persistence in tissues and host susceptibility factors results in the development of joint inflammation (see Figure 1). Diagnosis is impaired by delayed presentation and difficulties in microbe identification. Outlook in these patients is generally favourable although they remain at higher risk of developing spondyloarthropathies.

## Figures and Tables

**Box 1:**
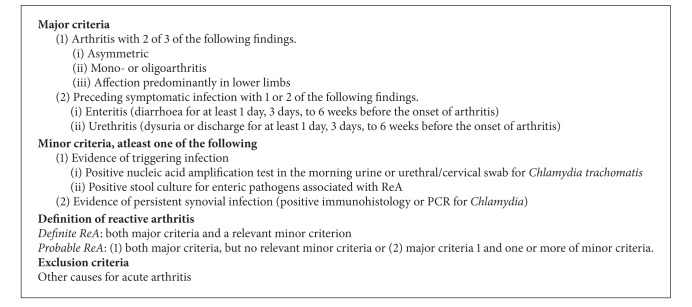
Preliminary classification criteria for reactive arthritis (adapted from Braun et al. [3]).
